# Purpuric Rash and Thrombocytopenia After the mRNA-1273 (Moderna) COVID-19 Vaccine

**DOI:** 10.7759/cureus.14099

**Published:** 2021-03-25

**Authors:** Srikrishna V Malayala, Gisha Mohan, Deepa Vasireddy, Paavani Atluri

**Affiliations:** 1 Internal Medicine, Temple University Hospital, Philadelphia, USA; 2 Medical Research, Physicians of American Healthcare Access, Philadelphia, USA; 3 Pediatrics, Pediatric Group of Acadiana, Lafayette, USA; 4 Internal Medicine, Bay Area Hospital, Coos Bay, USA

**Keywords:** covid 19, immune thrombocytopenia purpura, drug-induced itp, mrna-based vaccine, drug rash

## Abstract

The mRNA-1273 vaccine, popularly called the "Moderna vaccine" is being widely administered in the United States for the prevention of COVID-19 infection since December 2020. Mild to moderate intensity side effects like low-grade fever, myalgia, chills and malaise were reported in the trials related to the vaccine. With this case report, we report a case of purpuric rash and thrombocytopenia after receiving the first dose of the m-RNA-1273 vaccine. The patient, in this case, is a 60-year-old male patient who received the first vaccine dose and within two days, he developed diffuse papular rash associated with some thrombocytopenia. He had a history of tobacco use, Hepatitis C liver cirrhosis, chronic kidney disease stage 4, untreated hypertension and systolic congestive heart failure at the baseline. With review of the limited literature related to the vaccine and its side effect profile and with no other etiology explaining the sudden onset of rash, we attribute this thrombocytopenia and purpuric rash as the side effects of the mRNA-1273 vaccine.

## Introduction

The COVID-19 pandemic led to 117 million infections and 2.6 million deaths worldwide by March 2021 [1]. This called for an urgent need for vaccinations against COVID-19 and almost 120 different SARS-CoV-2 vaccines were being manufactured by the end of 2020 [2]. The mRNA-1273 vaccine, popularly called the "Moderna vaccine" is a nucleoside-modified messenger RNA (mRNA)-based vaccine that has been widely administered in the United States since December 2020 [3]. In the clinical trials of the vaccine, the side effects typically happened within seven days of getting vaccinated but the severity of these side effects were mostly mild to moderate. Low-grade fever, myalgia, chills, malaise and headache were more common after the second dose of the vaccine. A very small number of people reported severe side effects that required active management or even hospitalization [4]. 

We report a case of purpuric rash and thrombocytopenia after the first dose of the mRNA-1273 vaccine. This is a case report of a 60-year-old male patient who received the first vaccine dose and within two days, he developed diffuse papular rash associated with thrombocytopenia.

## Case presentation

The patient is a 60-year-old African American male patient who presented to the emergency room with multiple symptoms including generalized weakness, shortness of breath, leg edema, nausea, vomiting, abdominal pain, chest pain and generalized rash as the presenting symptoms in the first week of March 2021.

He is a resident of Philadelphia, Pennsylvania, USA. At the baseline, patient had a history of Hepatitis C infection in the past when he was incarcerated. He was treated with Interferon and he claimed himself "cured out of Hepatitis C." He carried a history of chronic kidney stage 4 with a baseline creatinine of 3 mg/dl. He also had a history of untreated systemic hypertension and systolic congestive heart failure with an ejection fraction 35%. He was not taking any medications for any of his co-morbidities and did not have a primary care physician. He was an active smoker, currently smoking 1 pack of cigarettes per day. He did not receive the influenza vaccine this year. 

Two days prior to the admission, he received the first dose of COVID-19 mRNA 1273 (Moderna) vaccine. A day after receiving the vaccine, he started experiencing these “flu-like” symptoms including low-grade fever, chills, nausea and few loose stools. He also developed a generalized rash a day after receiving the vaccine. He was tested negative for COVID-19 in February 2021. This was tested as a part of routine screening in his previous admission when he presented with congestive heart failure exacerbation but left the hospital against medical advice without completing the treatment and work up.

On presentation to the emergency room, he was afebrile, had a blood pressure 183/106 mm Hg, pulse rate of 82 per minute and oxygen saturation of 96% on room air.

On examination, he had wet crackles in both the lung bases, pitting pedal edema in both the lower extremities and generalized abdominal distention without any tenderness.

**Figure 1:**
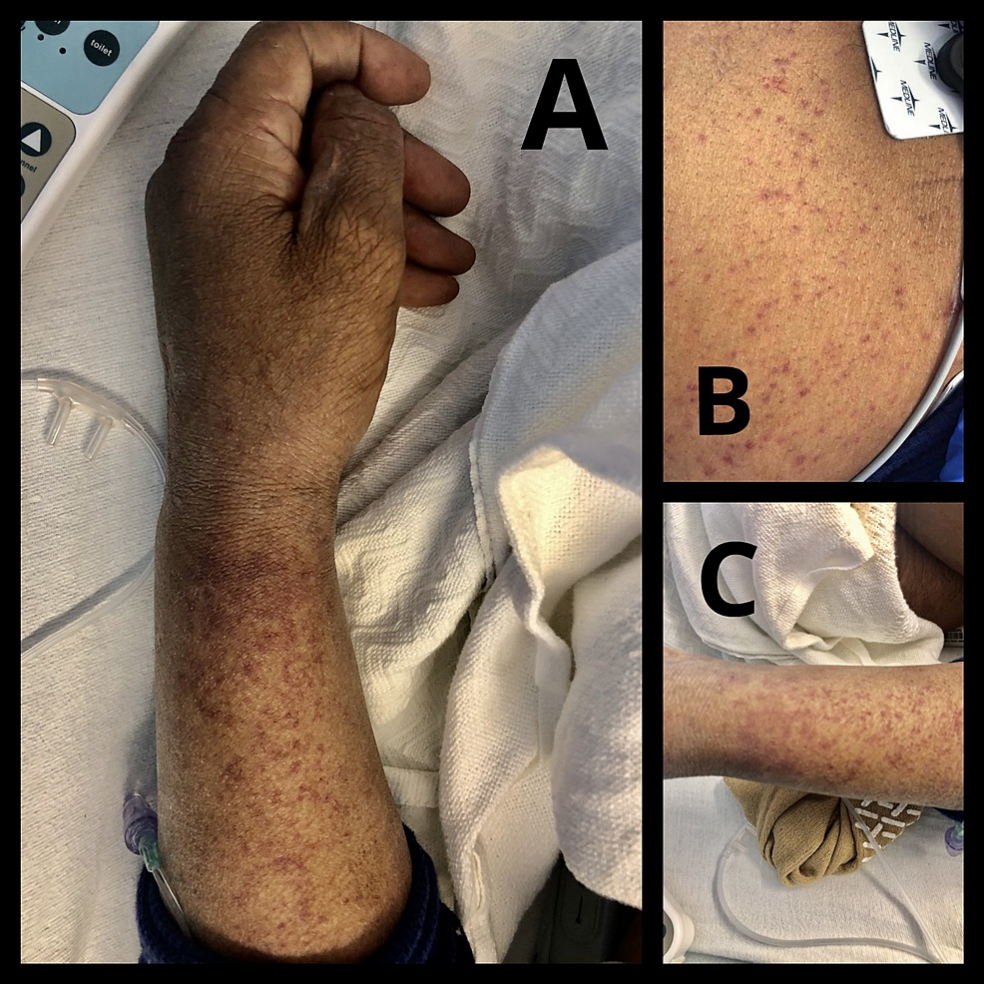
Papular rash on the left forehand (A), chest (B) and left leg (C) after receiving the COVID-19 mRNA-1273 vaccine.

The skin examination was consistent with a significant generalized rash. The rash was brown to red-colored, purpuric and not blanch-able. The rash itself or the area of the body was not tender to palpation (Figure 1). He also had a 5x5 cm bruise on the left shin. The rash was located all over the body, including the trunk, all the four extremities, predominantly on the forearms, shins and chest wall. On reviewing further history regarding the rash, he described the rash as a non-itchy, non-painful rash. It was sudden in onset, started the day after receiving the vaccine and quickly spread throughout the body. He denied nose bleeds, hemoptysis, hematemesis, joint pains or discoloration of stool. 

On admission, he was again tested for COVID-19 infection by PCR and was negative. Urinalysis showed 3 plus hematuria. Renal function panel showed a creatinine of 3.2 mg/dl, estimated glomerular filtration rate (eGFR) of 19. Hepatic function panel was quite abnormal with a AST level of 196 U/L, ALT level of 90 U/L, ALP level of 216 U/L, albumin 1.9 gm/dl and direct bilirubin 05 mg/dl. His hematology panel showed a low plateful level of 84,000/uL. INR was 1.13. The chest X-ray showed findings consistent with pulmonary edema. 

He was admitted to the hospital with a diagnosis of accelerated hypertension, systolic congestive heart failure exacerbation, abnormal liver functions that required workup and a purpuric rash. He was started on anti-hypertensives to get the blood pressure under control and intravenous diuretics to treat the heart failure exacerbation. 

After admission, he had an ultrasound of the liver that confirmed liver cirrhosis. Hepatitis panel showed a heavy viral load of more than 11 (reference range s/co ratio). Rest of the hepatitis panel and HIV were reported negative. Influenza A and B were reported negative. Blood cultures were reported negative.

Ferritin level (checked as a part of "inflammatory" panel) was elevated to 2400 ng/ml (reference range: 24-336 ng/ml), LDH was elevated to 381 U/L (reference range: 122-222 U/L) and CRP was normal at 5 mg/L (reference range: 0-8 mg/L). Anti-myeloperoxidase (MPO), anti-proteinase 3, p-ANCA, c-ANCA antibody levels were within normal limits. C3 and C4 complement levels were also within normal limits. 

With the ongoing management, his liver and renal function panels were stable but the platelet level continued to drop daily. We formulated to plan to monitor the platelet level, perform further platelet function tests, maintain further negative fluid balance, titrate anti-hypertensives for optimum blood pressure management and eventually conduct ischemic evaluation to establish the etiology of congestive heart failure. We also referred him to a gastro-enterologist to manage his newly diagnosed liver cirrhosis and resume management of Hepatitis C. However, on day 3 of hospitalization, patient left the hospital against medical advice without taking any of his prescriptions or following further instructions.

Notably, his platelet count was within normal limits two weeks prior to this admission (155,000/ul). 

## Discussion

We report a case of thrombocytopenia and related purpuric rash which are possibly the side effects of COVID-19 mRNA-1273 vaccination. Given the sudden onset of symptoms, absence of previous similar episodes, short latency period following vaccination and excluding other causes of the same, the patient might have developed these symptoms following the vaccine. With this manuscript, we intend to discuss the current literature related to the mRNA vaccine-induced rash and the pathogenesis of thrombocytopenia related to the COVID-19 vaccines. 

Immune thrombocytopenia is an autoimmune disorder, where one's own immune system attacks the circulating platelets and platelet production. It can be primary and secondary. The most common cause of immune thrombocytopenia is idiopathic, but there are secondary causes like infection, immunodeficiency, autoimmune diseases and vaccination resulting in platelet destruction, thrombocytopenia leading to mucocutaneous and major bleeding [5].

We attribute the purpuric rash and the thrombocytopenia in this case could be induced by the vaccine. Immune thrombocytopenic purpura (ITP) has been associated with several vaccinations like measles, mumps, rubella (MMR), hepatitis A, varicella, diphtheria, tetanus, pertussis (DPT), oral polio and even influenza vaccines in both children and adults [6]. A well-known association between the influenza vaccine and ITP is based on retrospective analysis that describes this association [6]. According to the WHO (World Health Organization) standardized causality assessment, it was concluded influenza vaccinations associated with ITP with an odds ratio of 3.8 (95% confidence interval 1.5-9.1) [3,6]. There are also case reports published describing vaccination-related ITP and increased platelets associated IgG (pAIgG) antibodies following the same, further strengthened by resolution with corticosteroid treatment [7,8]. The MMR vaccine’s association to thrombocytopenia demonstrated one to three children developing ITP for every 100,000 vaccine doses [9,10].

Even though the pathogenesis of vaccine-related ITP is not clear, the vaccines could lead to ITP by molecular mimicry, a process by which peptides in vaccines which are similar to platelet antigens resulting in activation of autoreactive B or T cells. It can result in elevated PAIgG, epitope spreading, and polyclonal activation that might be leading to ITP following vaccination [6,11-13]. Sometimes the adjuvants present in vaccines are said to be responsible for auto-antibody activation and immune response, termed as autoimmune/inflammatory syndrome induced by adjuvants (ASIA) [14].

General treatments of immune thrombocytopenia include medical treatments such as corticosteroids, intravenous immunoglobulin (IVIG) and IV Rh anti-D. Second-line pharmacotherapy such as dapsone and rituximab are also commonly used. In case of severe life-threatening hemorrhage, surgical intervention like splenectomy is suggested [15]. ITP secondary to vaccination can vary from mild to a severe intensity, but there is a high responsiveness to intravenous immunoglobulin (IVIg) treatment in severe cases [16].

COVID-19 vaccines had been reported to have mild to moderate side effects that can be managed symptomatically. This is the third case reported to have thrombocytopenia and purpuric rash apart from the press reports [17,18]. The rash after receiving the vaccine has been popularly described as “COVID arm” in news media which is a delayed reaction that causes a red, and sometimes bumpy rash on the arm. These reactions usually occur within 48 hours of receiving the vaccine, but in some cases, the rash was seen even 8 to 10 days after receiving the vaccine [19].

When dealing with a patient with ITP, it is important to cover the history of recent infections, childhood history, medications and vaccination history to rule out the differential diagnosis [20]. The differential diagnoses considered in this patient were thrombocytopenia related to cirrhosis, Hepatitis C and other autoimmune and infectious etiologies. However, the fact that the platelet count was within normal limits two weeks prior to the vaccine ruled out chronic bone marrow suppression related to cirrhosis. HIV, influenza, COVID-19 infection were ruled out and the rest of the auto-antibody panel were also reported negative.

## Conclusions

The literature regarding the side effect profile of COVID-19 vaccines is extremely limited. With an exception of an angry red rash being called “COVID arm” in new media, there is no peer-reviewed scholarly literature related to any of the COVID-19 vaccines. We attribute the purpuric rash in our case as a side effect of the m-RNA 1273 vaccine; though it could have been precipitated by the underlying thrombocytopenia, liver cirrhosis and Hepatitis C infection. With widespread distribution and administration of COVID-19 vaccine, we could anticipate more data regarding the side effect profile of the COVID-19 mRNA and other vaccines and the mechanism and pathophysiology of such side effects. The safety of the mRNA COVID-19 vaccines with underlying history of hepatitis, liver cirrhosis and thrombocytopenia also needs to be investigated with future studies. COVID-19 vaccines reportedly have very few side effects and in the background of benefits over risks from the vaccine, this article should not alter the recommendations to take the vaccine. Nevertheless, we wish to alert the clinicians to this possible association of ITP post-vaccination.

## References

[1] (2021). COVID-19 Coronavirus Pandemic. https://www.worldometers.info/coronavirus/.

[2] (2021). The COVID-19 candidate vaccine landscape and tracker. https://www.who.int/who-documents-detail/draft-landscape-of-covid-19-candidate-vaccines.

[3] Jackson LA, Anderson EJ, Rouphael NG (2020). An mRNA vaccine against SARS-CoV-2 - preliminary report. N Engl J Med.

[4] Baden LR, El Sahly HM, Essink B (2021). Efficacy and safety of the mRNA-1273 SARS-CoV-2 vaccine. N Engl J Med.

[5] Cines DB, Bussel JB, Liebman HA, Luning Prak ET (2009). The ITP syndrome: pathogenic and clinical diversity. Blood.

[6] Perricone C, Ceccarelli F, Nesher G (2014). Immune thrombocytopenic purpura (ITP) associated with vaccinations: a review of reported cases. Immunol Res.

[7] George JN (1990). Platelet immunoglobulin G: its significance for the evaluation of thrombocytopenia and for understanding the origin of alpha-granule proteins. Blood.

[8] Nagasaki J, Manabe M, Ido K (2016). Postinfluenza vaccination idiopathic thrombocytopenic purpura in three elderly patients. Case Rep Hematol.

[9] Cecinati V, Principi N, Brescia L, Giordano P, Esposito S (2013). Vaccine administration and the development of immune thrombocytopenic purpura in children. Hum Vaccin Immunother.

[10] France EK, Glanz J, Xu S (2008). Risk of immune thrombocytopenic purpura after measles-mumps-rubella immunization in children. Pediatrics.

[11] Guimarães LE, Baker B, Perricone C, Shoenfeld Y (2015). Vaccines, adjuvants and autoimmunity. Pharmacol Res.

[12] Kivity S, Agmon-Levin N, Blank M, Shoenfeld Y (2009). Infections and autoimmunity--friends or foes?. Trends Immunol.

[13] Pordeus V, Szyper-Kravitz M, Levy RA, Vaz NM, Shoenfeld Y (2008). Infections and autoimmunity: a panorama. Clin Rev Allergy Immunol.

[14] Pellegrino P, Clementi E, Radice S (2015). On vaccine's adjuvants and autoimmunity: current evidence and future perspectives. Autoimmun Rev.

[15] Warrier R, Chauhan A (2012). Management of immune thrombocytopenic purpura: an update. Ochsner J.

[16] Black C, Kaye JA, Jick H (2003). MMR vaccine and idiopathic thrombocytopaenic purpura. Br J Clin Pharmacol.

[17] Weintraub K (2021). Death of Florida doctor after receiving COVID-19 vaccine under investigation. https://www.usatoday.com/story/news/health/2021/01/06/death-florida-doctor-following-pfizer-covid-19-vaccine-under-investigation-gregory-michael/6574414002/.

[18] Tarawneh O, Tarawneh H (2021). Immune thrombocytopenia in a 22-year-old post Covid-19 vaccine. Am J Hematol.

[19] (2021). 'COVID arm': Some develop delayed skin reaction after Moderna vaccine shot. https://abc7ny.com/covid-vaccine-reaction-side-effects-arm/10398907/.

[20] Mohan G, Malayala SV, Mehta P, Balla M (2020). A comprehensive review of congenital platelet disorders, thrombocytopenias and thrombocytopathies. Cureus.

